# Baseline and Dynamic Expression of Activating NK Cell Receptors in the Control of Chronic Viral Infections: The Paradigm of HIV-1 and HCV

**DOI:** 10.3389/fimmu.2014.00305

**Published:** 2014-07-02

**Authors:** Francesco Marras, Federica Bozzano, Maria Libera Ascierto, Andrea De Maria

**Affiliations:** ^1^Istituto Giannina Gaslini, Genova, Italy; ^2^Center of Excellence for Biomedical Research, University of Genova, Genova, Italy; ^3^Department of Experimental Medicine, University of Genova, Genova, Italy; ^4^Department of Oncology, Johns Hopkins University, Baltimore, MD, USA; ^5^Department of Health Sciences, University of Genova, Genova, Italy; ^6^Clinica Malattie Infettive, IRCCS A.O.U. S. Martino-IST, Istituto Nazionale Ricerca sul Cancro, Genova, Italy

**Keywords:** natural cytotoxicity receptors, NKp46, NKp30, HIV, HCV, regulation

## Abstract

Natural killer (NK) cell function is regulated by a balance between the triggering of activating and inhibitory receptors expressed on their surface. A relevant effort has been focused so far on the study of KIR carriage/expression setting the basis for NK cell education and self-tolerance. Focus on the evolution and regulation of activating NK receptors has lagged behind so far. Our understanding of activating receptor expression and regulation has recently improved by evidences derived from *in vitro* and *in vivo* studies. Virus infection – either acute or chronic – determines preferential expansion of NK cells with specific phenotype, activating receptors, and with recall-like functional activity. Studies on patients with viral infection (HIV and HCV) and specific diverging clinical courses confirm that inter-individual differences may exist in baseline expression of natural cytotoxicity receptors (NKp46 and NKp30). The findings that patients with divergent clinical courses have different kinetics of activating receptor density expression upon NK cell activation *in vitro* provide an additional, time-dependent, functional parameter. Kinetic changes in receptor expression thus represent an additional parameter to basal receptor density expression. Different expression and inducibilities of activating receptors on NK cells contribute to the high diversity of NK cell populations and may help our understanding of the inter-individual differences in innate responses that underlie divergent disease courses.

## Introduction

Natural killer (NK) cells represent a cellular component of the innate immune system. They circulate in peripheral blood and peripheral tissues, and may be more abundantly recovered in secondary lymphoid organs and in some non-lymphoid organs (e.g., the liver). They are characterized by considerable cytotoxic activity, which is due to the constitutive expression of perforin and granzyme, which may be promptly released upon cell triggering (1, 2). In addition to this unique feature, which contributes to the high efficiency with which NK cells are suited to kill virally infected or tumor cells, their function also includes the production of cytokines, such as IFN-γ, tumor necrosis factor (TNFα) and G-CSF, and the early release of chemokines (MIP-1a/b, RANTES) (3). NK cell function is finely regulated by the interplay of a wide array of activating and inhibitory receptors expressed on their surface (1). The accurate regulation of this signaling system guarantees that under baseline conditions their exceptional ability to rapidly kill is harnessed by inhibitory receptors mostly (but not exclusively, e.g., Siglec7 and IRP60) specific for “self” MHC class I molecules (2), which turn NK cells “off” and normally prevent NK-mediated lysis of HLA class I^+^ autologous cells.

Natural killer cell activating stimuli are manifold and act through triggering of different groups of receptors expressed on their surface (4). Activation of NK cells is determined by triggering the major activating receptors, which include NKp46, NKp30, NKp44 (i.e., natural cytotoxicity receptors, NCRs) NKG2D, and FcγR (CD16) as well as other receptors and co-receptors including, NKG2C (a lectin-type triggering receptor which dimerizes with CD94), 2B4 (CD244), NKp80, DNAM-1, and NTB-A (5). Stimuli delivered through other groups of receptors may also determine NK cell activation including toll-like receptors (TLRs) as TLR2, TLR3, TLR7/8, TLR9, and interleukin receptors (IL-2, IL-12, IL-15, IL-18) and combinations thereof (e.g., IL-2 + IL-15, IL-2 + IL-12, and IL-12 + IL-18) (5–7).

The NK cell receptors repertoire is germ line-encoded and does not undergo somatic recombination. This provides the basis for inclusion of NK cells among innate host defenses as an effective and apparently basic unsophisticated innate defense system which does not need specific recognition of foreign antigens (e.g., from pathogens or tumors).

## Involvement of NK Cells in the Tuning and Control of Immune Responses

The original view of NK cells as a purely “primitive,” muscular, short-lived, rapid responder cell type has undergone considerable revision and has been considerably updated over recent years. NK cells are presently known to represent long-lived innate cells, whose functional spectrum extends beyond classical search-and-destroy patrolling activity and/or early recruitment of immune responses. NK cell function indeed also includes regulation of other innate and adaptive functions through their direct or indirect reciprocal interaction (crosstalk) with macrophages, polymorphonuclear cells (6, 7), fibroblasts (8), DC (9–11), and T cells.

Natural killer cells interact with and respond to either pathogen-infected or tumor-associated macrophages, and their response is modulated by macrophage functional polarization. The functional interaction of NK cells with pro-inflammatory (M1) or anti-inflammatory (M2) macrophages relies on DNAM-1, 2B4, and NKp46 receptor signaling, in addition to membrane-bound macrophage-derived IL-18 (12–14). Considering macrophages infected with specific pathogens, different sets of activating receptor–ligand interactions drive NK cell activation in a more pathogen-dependent pattern (6, 11, 15). A decreased NK cell response has been described during their interaction with M2 cells compared to M1 macrophages, or in the tumor environment where NK cell activating receptors are down-regulated (13, 14). In view of the reported recognition of mycobacteria-infected macrophages by NK cells via NKp46 (15), decreased signaling via NKp46 in patients with overt secondary pulmonary TB (16) could represent for example a possible mechanism participating in the so far poorly understood mechanism of exit from latent TB (17).

The reciprocal interaction of NK cells and DC is often referred to as “crosstalk” and involves multiple receptors and cytokines. NK cells and DC show anatomical and functional co-localization in T cell areas of lymphnodes (18) and in inflamed tissues (19). Human DC activate NK cells, via direct interaction and involvement of NKp30 (10, 20) and DNAM-1 (9). NK cells determine lysis of immature HLA class I^−/low^ DC, while mature DC are protected from NK-mediated lysis by high density expression of HLA class I molecules that interact with inhibitory receptors (e.g., KIR) expressed on activated NK cells (11). In turn, NK cells induce DC maturation via TNFα and IFNγ production (10). This DC–NK cell interaction provides a mechanism to edit DC responses and their repertoire by selecting optimally mature DCs (21) and inducing DC to express high amounts of membrane-associated IL-15 (22) with impact on downstream adaptive responses (23). These observations are further supported by the demonstration, *in vitro* with bacterial infection (24) and *in vivo* during HIV infection that the presence of reduced NCR expression (25) and NK cell subset alterations (26) leads to reduced killing of immature DC (20).

Involvement of NK cells in the shaping of adaptive responses extends beyond their crosstalk with DC. In fact, NK cells also directly interact with T cells, favoring antigen-specific CD8^+^ T cell responses (22, 23, 27). The reciprocity of this circuit has been elegantly shown in the macaque model where Ag-specific CD4^+^ T-central memory lymphocytes support NK cell activation and function in SIV-controller donors (28).

Therefore, a relevant part of the tuning activity of NK cells on the function and control of other cells is based on direct cell-to-cell interactions and involves activating NK cell receptors. Consequently, different activating NK cell receptor molecule densities may have an impact on their crosstalk with other cells of the immune system. The purpose of this review is to provide a reading frame to differences in static and dynamic NCR expression in subjects displaying clinical divergence upon infection with different viruses.

## Innate or Not Innate, This is the Question

Until recently, the prevailing expert view attributed to NK cells a limited degree of variability in response to pathogens, and basically assumed stereotyped responses. This concept practically ruled out the possibility of ranges of variability of NK cell responses either against different pathogens within the same subject or to the same pathogen within different patients. This view has been steadily upgraded in recent years. It has been shown for example in mice that infection with viruses and other pathogens determines the expansion of specific NK cell subsets (29–31) which maintain for prolonged periods of time the ability to produce increased amounts of TNFα and IFNγ. This observation is reminiscent of memory T cell function thus suggesting a possible memory-like feature of NK cells. Subsequent observations in human beings showed that also human CMV infection leads to expansion of a subset of NKG2C^+^ NK cells (32, 33) with memory-like properties. Increased proportions of NKG2C^+^ NK cells persist (34) after acute infection into latency, and may be observed also after bone marrow transplantation (34, 35). Additional evidences of transient NK cell expansions in human beings are provided by infection with chikungunya and hantavirus (36, 37), and may persist up to 60–90 days. Also in these instances the expanded cells are exclusively NKG2C^+^, and their triggering results in increased and rapid reactivity with production of IFNγ upon re-challenge. These observations are clearly different from T cell memory, which is conventionally defined by (life)long-lasting antigen-specific recall ability, increased memory T cell receptor (TCR) density, antigen-specific TCRs, and specific markers identifying memory cells (CD45RO vs. CD45RA) (31, 38). In mice, LY49H^+^-MCMVm157 antigen-specificity and increased protection to Mouse CMV (MCMV) challenge support a resemblance with memory T cell protection (31, 39). In human beings, on the other hand, the expansions are more time-limited with the possible exception of HCMV infection and latency. These human NK cell subset expansions are stereotyped and monomorphic since only NKG2C^+^ cell expansions are reported, irrespective of the invading pathogen (either HCMV, or hantavirus or chikungunya).

Altogether, the description of pathogen-induced recall NK cell reactivity, even if not fitting with a long-lasting heterogeneous T cell memory (40, 41), has the merit of further expanding our understanding of NK cell function advancing our view beyond the original “first-line of defense” towards a wider horizon of multifaceted NK cell function.

An interesting contribution to the notion of a polymorphism in NK cell responses has been recently provided by mass cytometry study of NK cell receptor carriage (42) showing that up to 30,000 different phenotypic NK cell populations may be harbored in one individual. In addition, environmental factors appear to heavily influence activating receptor carriage, while inhibitory receptor diversity seems to be largely genetically determined (42). In line with this observation, NK cell responses induced by virus(es) are accompanied, not only by expansion of a subset of peripheral NK cells which may be NKG2C^+^ (33), but also by changes in triggering receptor expression (e.g., NCRs) (34).

## NCR Expression and HCV Infection

Natural killer cell triggering receptors recognize specific ligands induced by either cell transformation or infection [e.g., B7-H6 (41, 43)]. Although cellular ligands for NCRs are still not yet well characterized (e.g., NKp46-L, NKp44-L, and other NKp30-ligands), a range of pathogens including influenza virus, parainfluenza virus, West Nile virus (WNV), dengue virus, and mycobacteria have been shown to interact with NCRs, either directly or after their infection of target cells (44). Accordingly, NK cell first-line defenses against pathogens and regulation of immune responses may be more intertwined than expected at a first look. Indeed, the same receptors that are involved in recognition of infected cells may be also involved in direct pathogen detection and in crosstalk with other cells of the immune system (e.g., monocytes and dendritic cells). Hence, individual differences in baseline NCR expression may underlie and affect divergent host responses to the pathogen.

Natural killer cell triggering is proportional to both the actual number/density of a given triggering receptor expressed on NK cells and to the density of the respective ligand(s) expressed on target cells. At any given level of KIR/HLA interaction and of NK-ligand expression, changes in activating receptor expression density determines proportional changes in NK cell cytotoxic activity(45, 46).

Accordingly, wide inter-individual variations in triggering receptor expression could contribute, at least in part, to the different clinical courses (e.g., from mild disease to life-threatening clinical course) that are observed in patients infected by the same pathogen. When for example considering NKp46 and influenza hemagglutinin (HA), NKp46 (and NKG2D) is necessary for the activation of the human response to influenza infection (47, 48). In mice, induced deletion of the human NKp46 homolog (NCR1) determines lethal influenza infection (49). In addition, NK cell function is impaired in aging mice infected with influenza virus, with reduced production of IFN-γ also upon stimulation with anti-NKp46 mAbs, in line with the suggestion of reduced receptor expression (49, 50).

The possibility that different levels of NCR expression may correlate to different clinical courses emerges also from studies in patients with HCV infection. A relevant association exists between outcome of acute HCV infection and germline carriage of KIR genes and HLA C supertypes (51, 52). Also activating NK cell receptor expression has been shown to associate with different disease courses during HCV infection. In the acute phase of HCV infection, an increase in CD56^bright^ NK cells is observed, and is accompanied by a reciprocal reduction in CD56^dim^ cells (53). In patients who spontaneously clear the virus (HCV), the increase in CD56^bright^ NK cells is transient, with subsequent decline within 1–3 months. This change is however permanent in those who fail to clear HCV and proceed to chronic infection with virus replication (53). Interestingly, NKp30 expression is increased in NK cells from multiply HCV exposed-uninfected intravenous drug users. In these patients, enhanced IL-2-induced cytolytic activity against the NK-sensitive cell line K562 has been also reported (54). The same mechanism of NKp30-associated protection applies to the control of *in vitro* hepatocyte infection (54). In a recent and different setting, increased NKp46, NKp44, and NKG2A expression was detected in NK cells from HCV exposed healthcare workers (HCW), who did not develop disease (55). Interestingly, in this series, HCV-specific T cell responses to non-structural gene products were detected in the absence of B cell responses and of HCV-specific antibody production. This observation is reminiscent of a phenomenon occurring in HIV-uninfected children born to HIV-seropositive mothers. These children lose maternal antibodies are uninfected and seronegative, but show HIV-specific cytotoxic activity by CD8^+^ T cells (56, 57). An analogy is evident in results obtained 20-years apart in different models of human infection and raises the possibility that inducibility of NCRs on NK cells in some patients may be associated with discordant T and B cell responses and with protection from infection.

Differences in NCR expression are detected also when exposed patients become acutely infected and display HCV viremia. In this case, lower frequencies of NKp46- and NKp30-expressing NK cells are observed compared to healthy donors, and this phenotype correlates with HCV clearance (58). Thus, NCR (NKp46 and NKp30) expression on peripheral NK cells is different when acutely infected patients (low NCRs) are compared to exposed-uninfected patients (high NCRs). The observed difference may be for example due to inherent, preexisting baseline differences that become evident following patient selection in different series. Alternatively, in patients who resolve acute infection, the lower expression of NKp46- and NKp30 molecules on NK cells could be due to increased margination of NK cells to the site of active HCV replication (i.e., liver) (59). A further possible explanation for these differences could be represented by different inductions of NCR expression in different patient groups upon challenge with the virus (HCV). In such a scenario, early responders would rapidly upregulate NCR expression, avoid establishment of infection, and evolve to the clinical state of exposed-uninfected subjects.

During chronic HCV infection, imbalances in peripheral NK cells were originally described, with a deficient ability to activate DCs due to the interaction of NKG2A with HLA-E expressed on hepatocytes (60). This interaction is associated to IL-10 production (60, 61) and to reduced ability of IFNγ production upon IL-12 stimulation or with other stimuli (62). With regard to NCR expression, either increases or decreases in NCR expression are observed (61, 63) and are related to their ability to respond to IFN-α-containing treatment regimens. Indeed, patients who clear the infection upon dual treatment with pegylated-IFN-α + ribavirin (PegIFNα–ribavirin) have lower baseline (pre-treatment) expression of NKp30 and of CD85j, compared to those who subsequently fail the treatment (null responders, partial responders) (64). The decreased expression of NKp30 in these patients is not reflecting a defect in NK cell function, but rather an individual difference in the regulation of receptor expression. This is confirmed by the successful induction *in vitro* of NKp30 expression on purified NK cells cultured in the presence of IFN-α, with correspondingly increased receptor-mediated function. These observations are in line with evidences deriving from the study of intrahepatic interferon-stimulated gene (ISG) expression (65, 66). In the liver, ISG is already upregulated before treatment in patients who will not respond to IFN-α and ribavirin dual treatment. Patients who will clear virus upon treatment, on the contrary, have baseline lower ISG expression which may be induced during treatment (66). Thus, inherent individual regulation of crucial gene expression is present during chronic HCV infection and extends from ISG in hepatocytes to NCRs.

Therefore, differences in baseline NCR expression and in NK cell phenotype can be accurately detected (42) and are associated with diverging clinical courses in subjects exposed to HCV. Lower NCR expression, albeit inducible, represents an advantage and appears to be inherently regulated in a subset of chronically infected patients. In this context, the increased NCR expression observed on NK cells in exposed-uninfected patients may be the result of repeated HCV challenge in patients with lower baseline NCR expression.

## NCR Expression and Diverging Clinical Courses in HIV Infection

During HIV infection, the virus targets NK cell-mediated responses with similar or possibly higher intensity compared to other arms of innate or adaptive immunity. NK cells are part of the early response that controls acute viremia during primary HIV infection. Similar to HCV exposed-uninfected patients (54, 55), increased NK cell activity has been detected also in HIV exposed-uninfected patients (67). Activating receptor expression was not addressed in this work, and materials for monitoring (anti-NCR mAbs) were not yet available at the time. Although the mechanisms leading to increased NK cell function in those HIV exposed-uninfected remain still not defined, increases in the expression of triggering receptors on NK cells could have been possible, similar to what has been observed after exposure to HCV (54, 55). If so, this would fit in a broad concept of an advantage against infection in subjects whose NK cells achieve rapid dynamic increases in NCR expression after virus challenge.

Once HIV viremia is established, NK cell derangement can be detected in infected patients soon thereafter (68–70) and includes imbalances in activating and inhibitory receptor expression, altered circulation of NK cell subsets, and impairment of NK cell function (25, 26, 71, 72). Viremic HIV-patients have dramatic decreases in activating receptor expression (NKp46 and NKp30) on NK cells *in vivo* (25), up to one-third of circulating NK cells display activation markers (HLA-DR and CD69) (72) and an apparently poorly functional subset of CD56^−^CD16^+^ appears, which displays expression of low levels of NCRs (26, 72). The impairment of NK cell function has a relevant impact on DC editing. In addition to the reduced activating receptor expression on NK cells and appearance of poorly functional CD56^−^CD16^+^ NK cells in peripheral blood, HIV infection reduces the expression on CD4^+^ T and other target cells of ligands of activating NK cell receptors which are important in triggering NK cell cytotoxicity and cytokine production (e.g., PVR, NKp46-L, and NKp30-L) (73–76). Thus, while HIV adopts multiple strategies to evade NK cell surveillance, conserved (or restoration of) activating receptor function may represent a fundamental barrier to virus spread.

From a clinical standpoint, during the first years of the epidemic, it became soon clear that consistent clinical variability could be observed among patients even in the absence of successful treatment. A benign disease course in otherwise untreated patients was identified through the observation of HIV-infected patients with long-term non-progressing disease (i.e., high CD4^+^ T cells >450/μl and low level viremia, LTNP) and of patients with high CD4^+^ T-lymphocytes and undetectable viremia (elite controller, EC) (77, 78). In addition, upon progressive disease with decreasing CD4^+^ cell counts, diverging courses are observed. At any given CD4^−^ count, opportunistic infections or cancers may appear in some – but not all – patients (79–81) and AIDS-defining disease including Kaposi Sarcoma, non-Hodgkin Lymphoma, or Tuberculosis may occur before CD4^+^ counts fall below 200–350/μl (82–85). On the other hand, in the pre-highly active antiretroviral treatment (HAART) era, some untreated patients could reach very low CD4^+^ T cell counts without progressing to AIDS and showed surprisingly preserved NK cell numbers and function (86). Following HAART, NK cells fail to fully recover IFN-γ production and phenotype (87, 88) and surprisingly maintain high levels of activation, as defined by HLA-DR expression (46, 89). This extensive list of examples shows the profound impact of personal clinical divergence in everyday HIV clinical practice.

Divergent clinical courses during HIV infection cannot be fully accounted for by CD4^+^ cell numbers alone. Data from NK cell function and receptor expression may be used in this context to help understand existing differences. When antiretroviral treatment is interrupted in chronically infected patients, viral replication invariably resumes even after thorough and extensive treatment (90). Trials of CD4^+^-guided treatment interruptions (CD4GTI) in patients with high CD4^+^ T cell showed that the rate of CD4^+^-cell loss after discontinuation of antiretroviral treatment is dishomogeneous with ample divergence and inter-individual differences (91, 92). Factors associated with different rates of CD4^+^ T cell loss upon CD4GTI include not only proviral DNA changes (93), increased proportion of CD4^+^CD127^+^ cells (94, 95), but also a different baseline NK cell expression and function (96). At baseline (i.e., before treatment interruption), patients with long treatment interruptions (i.e., without the need of resuming HAART) due to persistently high CD4^+^ cell counts had lower proportions of CD56^bright^CD16^±^ NK cells, and also expressed lower levels of NKp30 and NKp46 activating receptors on NK cells (96). Interestingly, this observation is in agreement with the findings from chronically HCV-infected patients, where those who will clear the virus after PegIFNα–ribavirin treatment have low – and inducible – NCR (NKp30) expression on their NK cells at baseline (64).

In addition, inherent differences in NK cell receptor expression are observed also in HIV-patients with low CD4^+^ cell counts (<220/μl) but divergent clinical course. Those who have AIDS-defining opportunistic infections (i.e., PCP and neurotoxoplasmosis) have lower NKp46 expression and low DNAM-1/NKG2D/NCR:ligand ratios compared to patients who reach similarly low CD4^+^ cell counts but do not develop AIDS (46). These results therefore indicate that similar to HCV patients, differences in NK cell regulation underlie divergent clinical courses also in HIV-patients, irrespective of their CD4^+^ T cell count, of HAART or virus replication. Importantly, this view is not limited to chronic infections. Support to the general view of inherent specific NK cell signatures underlying divergent disease course is provided also from analyses in cancer patients with recurrent disease, either gastrointestinal stromal tumor (GIST) (97) or breast cancer (98).

## Inducible NCR Expression and Clinical Divergent Disease Courses

Although analysis of NCR expression on peripheral NK cells may reflect baseline regulations in NK cell subsets, it represents only a “frozen” view and does not provide insights on their inducibility or “dynamic” regulation. In this regard, *in vitro* study of NCR expression in purified NK cells recently revealed that different induction kinetics over time may be detected in HIV-patients (99). Indeed, when patients with non-progressive disease course (EC and LTNP) were compared to HAART-treated aviremic patients, relevant differences were detected. In EC/LTNP patients, purified NK cells display increased NKp46 expression 2 days after *in vitro* activation with IL-2, with subsequent return to baseline expression 4 days thereafter. Also NKp30 expression is induced in EC/LTNP upon *in vitro* activation with progressive increased densities until 4 days after activation. On the contrary, HAART-treated progressor patients with undetectable HIV-RNA do not show any induction of NKp46 or NKp30 (99). The conserved induction of NCRs in non-progressor patients provides the basis for an intact NK cell function, conserved crosstalk with DCs and downstream specific CD8^+^ CTL responses (20, 100, 101). Interestingly, also in HCV-infected patients inducibility of NKp30 is associated with a different (improved) disease course compared to patients lacking this regulation (64). An additional observation of considerable interest in this context was the lack of inducibility of NKp44 in EC/LTNP patients compared to progressor patients (99). This apparent “fault” with failure to rapidly upregulate NKp44 molecule expression upon activation, might actually be highly protective once HIV infection has established. Indeed, HIV_gp41_ S3 peptide shedding in infected patients (102) induces expression of NKp44-ligands in uninfected CD4^+^ T cells. The apparent “faulty” induction of NKp44 thus would avoid innocent bystander killing of CD4^+^ NKp44-L^+^ cells by activated NK cells *in vivo*. Support to this hypothesis is directly provided by the demonstration that HIV_gp41_ vaccination prevents from shedding of the S3 peptide and from NKp44-L expression thus protecting CD4^+^ T_CM_-lymphocytes in SHIV-infected non-human primates (103).

A limitation to interpretation of NCR expression/induction is represented by the lack of molecular and genetic proof so far in the regulation of NCR responses and inducibility, with only limited information being available (104). Also, there is poor understanding of the mechanism(s) underlying “protection” from adverse disease in the presence of low-inducible activating receptor expression. It has been suggested that low-inducible NCR expression may provide advantage in the case of overwhelming stimulation of NK cells due to a relative preservation from their excessive activation with detrimental activation of other innate and adaptive immune mechanism(s) (44). In this regard, it has been shown that homo-oligomerization of NKp30 in the plasma membrane of NK cells is favored by IL-2-dependent up-regulation of NKp30 expression and improves recognition and lysis of target cells by NK cells (105). Interestingly, independent studies showed that viruses may address NKp30 receptors inactivating their function, as shown for NKp30 signaling by vaccinia virus HA (106). Thus, low expression of NKp30 with maintained inducibility, as in the case of HCV and HIV (64, 96, 99), could represent an advantageous inherent individual trait. Subjects carrying this characteristic would evade NK cell-targeting by virus(es) due to lower exposure of a given NCR (e.g., NKp30) to hyperactivation and inactivation (107). At the same time, they would still be able to upregulate molecule density upon activation (e.g., IL-2 and IFNα) thus reaching critical receptor homo-oligomerization with binding to its ligand (e.g., infected cells and DCs).

Evidences supporting memory-like NK cell responses and those showing inducibility of NCR receptor expression on NK cells are derived from clearly distinct settings and should be considered unrelated unless differently proven. Indeed, changes in cytokine production and in activating receptor expression have been reported during memory-like NK cell responses following CMV, hantavirus, or chikungunya virus infection (34, 36, 37), while NKp30 inducibility has been observed in some HCV or HIV infected patients (16, 96, 99). In addition, memory-like NK cell responses have been so far attributed only to NKG2C^+^ NK cells, while NCR inducible NK cell responses are largely NKG2C unrelated, and show a considerably shorter kinetic (2–4 vs. 30–60 days).

The concept of host antiviral NK cell-associated defenses may however be expanded to encompass both rapid inducibility of NK cell responses after virus infection and memory-like NK cell responses within the frame of NK cell diversity modulated by environmental factors (42). Diverging NCR inducibility, similar to memory-like NKG2C expansion both contribute to host protection and may represent different perspectives of a multifaceted ability of NK cells to adapt to tackle different invading pathogens.

## Conclusion

Manifold inter-individual differences in the regulation of innate defenses have been described until very recently. The immunogenotype of several components of innate immunity strongly influences the risk to contract infections and also the outcome of their treatment. Genome-wide association studies showed that polymorphisms of innate components are associated to individual variable response to treatment and to disease progression. Nucleotide polymorphisms of TLRs [TLR-4 (108, 109), TLR-1 and 6 (110)] and of pentraxin-3 (111) are associated to increased risk of invasive aspergillosis or mycosis in bone marrow transplant patients (110, 111). Similarly, TLR3 polymorphisms have been associated to pneumonia development in children with influenza virus infection (112). In addition, IL-28B single nucleotide polymorphisms are associated to prognosis of treatment in HCV infection using IFN-α-containing regimens (113–116).

**Figure 1:**
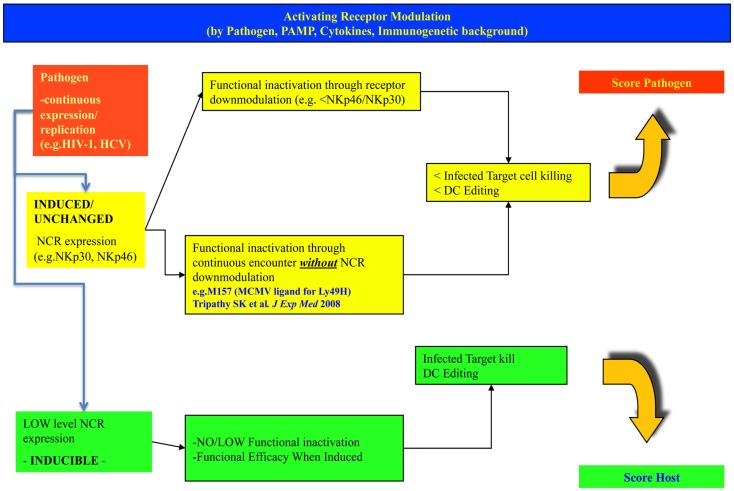
**Different levels and modulation of natural cytotoxicity receptors on NK cells may lead to different outcomes in the fight against invading intracellular pathogens**. The figure indicates possible alternate patterns of NK cell NCR expression/induction upon infection with a pathogen. In the upper row, the case for high basal expression is shown, leading to receptor inactivation and progressive infection. In the lower row, the case of a low/inducible NCR expression is shown, with inducibility upon strong challenge with successful control of the pathogen. The hypothesis considers a spectrum of intermediate conditions (not shown here).

**Figure 2:**
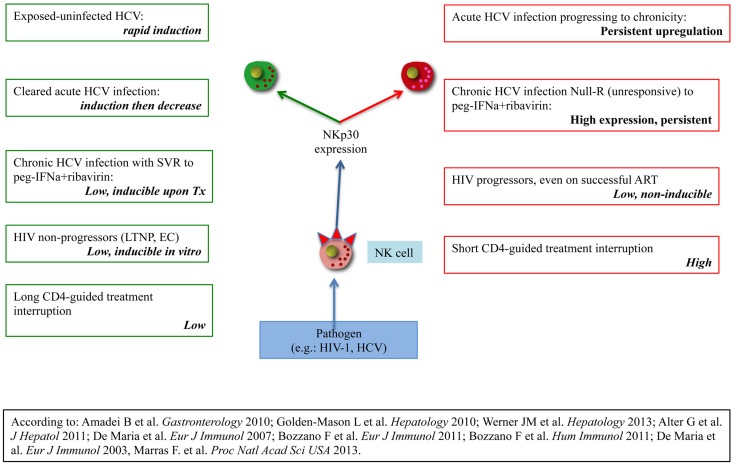
**Organization of evidences supporting clinical divergence in HIV-1 or HCV-infected patients according to NKp30 expression/regulation on peripheral NK cells**. The left column lists evidences showing favourable disease course in the presence of receptor inducibility. The left column lists evidences for unfavourable disease course in the presence of high basal expression without inducibility or no inducibility regardless of expression.

The presently discussed modulation of NCR expression (NKp30, NKp46, and NKp44) has been described also in chimpanzees (117), and thus appears to represent an innate mechanism of protection against chronic infections that is conserved in evolution and that provides inherent individual diversity in chronically infected (HIV and HCV) patients where it contributes to explain clinical divergence (Figures 1 and 2).

## Conflict of Interest Statement

The Guest Associate Editor Massimo Vitale declares that, despite being affiliated to the same institution as author Andrea De Maria, the review process was handled objectively and no conflict of interest exists. The authors declare that the research was conducted in the absence of any commercial or financial relationships that could be construed as a potential conflict of interest.
